# Considerations on the Demography of BIA-ALCL in European Countries Based on an E(A)SAPS Survey

**DOI:** 10.1007/s00266-021-02411-3

**Published:** 2021-07-20

**Authors:** Birgit Stark, Martin Magnéli, Ivar van Heijningen, Carlos Parreira, Urs Bösch, Michel Rouif, Martin Halle

**Affiliations:** 1 Department of Molecular Medicine and Surgery, Karolinska Institutet, Karolinska University Hospital, Stockholm, Sweden; 2 Institution for Clinical Sciences, Karolinska Institutet, Danderyd Hospital, Stockholm, Sweden; 3 Plastic Surgery Department, AZ Zeno and Duinbergen Clinic, Knokke-Heist, Belgium; 4 Hospital Garcia de Orta, Avenue Torrado da Silva, 2805-267 Almada, Portugal; 5 Clinica Corpuslaser, Av Estados Unidos da America, 100,1ºD, 1700-179 Lisbon, Portugal; 6 Plastic Surgery, MEON Clinic, Lucerne, Switzerland; 7 30, Bld Heurteloup, 37000 Tours, Loire Valley, France

**Keywords:** Breast implant-associated anaplastic large cell lymphoma, BIA-ALCL, EASAPS, European survey

## Abstract

**Background:**

A growing body of evidence indicates that breast implant-associated anaplastic large cell lymphoma (BIA-ALCL) is associated with the use of certain breast implants. Regional variations have been reported, and a genetic susceptibility has also been suggested. However, large variations in the ability to correctly diagnose BIA-ALCL and to further report and register cases exist between countries and may in part explain variations in the demography.

**Material and Methods:**

A survey was conducted by The European Association of Societies of Aesthetic Plastic Surgery E(A)SAPS and sent to 48 European countries. The primary aim was to identify the total number of confirmed cases of and deaths from BIA-ALCL in each country during four consecutive measurements over a two-year period.

**Results:**

An increase in BIA-ALCL cases during four repeated measurements from a total of 305 in April 2019 to 434 in November 2020 was reported by 23 of the 33 responding countries. A nearly 100-fold variation in the number of cases per million inhabitants was noted, where Netherlands had the highest rate (4.12) followed by Finland (1.99). Countries with the lowest reported rates were Austria (0.078), Romania (0.052) and Turkey (0.048).

**Conclusion:**

The current study displays a notable variation ßin the number of confirmed BIA-ALCL cases across Europe, even for countries with established breast implant registers. Variations in diagnosis and reporting systems may explain the differences, but the influence of genetic variations and the prevalence of high-risk implants cannot be excluded. Incomplete sales data along with medical tourism preclude an absolute risk assessment.

**Level of Evidence IV:**

This journal requires that authors assign a level of evidence to each article. For a full description of these Evidence-Based Medicine ratings, please refer to the Table of Contents or the online Instructions to Authors www.springer.com/00266.

## Introduction

Thirty-five Plastic and Aesthetic Surgery Societies are members of the European Association of Societies of Aesthetic Plastic Surgery (E(A)SAPS), initiated and founded by Ulrich Hinderer and Yann Levet in 2007. E(A)SAPS has identified 17 countries still without representation of plastic surgery in an independent professional society. Even though the main focus of E(A)SAPS remains consistent since its foundation, “serving European plastic surgeons and their societies with relevant scientific information,” the content of this focus became dramatically more complex as aesthetic surgery gained increasing attention in non-scientific dialogs in social media. Particularly the emergence of breast implant-associated anaplastic large cell lymphoma (BIA-ALCL) [1] and patient-reported breast implant illness (BII) [2] as well as the poly-implant prothesis (PIP) scandal [3] has shed new light on the important aspects of patient safety. A growing body of evidence indicates that BIA-ALCL is associated with the use of certain breast implants. BIA-ALCL was accepted by The World Health Organization (WHO) in 2016 and further included in the 2017 classification of hematolymphoid neoplasms [4–6]. Regional variations have been reported, and a genetic susceptibility also has been suggested [7]. The need for improved post-market surveillance of medical devices has been highlighted internationally thus providing the rationale for industry-independent breast implant registers [8]. The primary aim of the present study, conducted by the board of E(A)SAPS, was to compile European data on geographic distribution of confirmed cases and death rates of BIA-ALCL. Secondary aims were to evaluate the estimated prevalence of BIA-ALCL, reporting to national implant registers and identify countries that have implemented a ban on using textured breast implants.

## Material and Methods

### Survey

E(A)SAPS sent out surveys twice annually to 48 European countries from April 2019 until November 2020. Accumulated cases and deaths of BIA-ALCL are presented in Table 1. The questionnaires were completed by either the president or the secretary of the national society or a person nominated by the national society. The number of histopathologically confirmed BIA-ALCL cases and deaths in the specific country was recorded. The survey asked whether reporting of new cases of BIA-ALCL was done to notifying bodies, registers or other investigators and if this national reporting is mandatory. The existence of national breast implant registers and the accessibility to diagnostic centers for BIA-ALCL were further investigated. It was additionally assessed whether or not national health-care institutions banned the use of textured implants. The surveys were in accordance with European GDPR legal requirements. No specific data were collected or transmitted across country borders with potential track back to individual patients.

### European Populations

A total of 741.4 million people lived in the European countries by December 31, 2020 [9]. National sex ratios and the corresponding proportion of women between 15 and > 65 years for each country are presented in Table 1 [9]. This age range was chosen to cover the majority of eligible women with breast implants. In addition, reliable female data are internationally listed for this age range. The human sex ratio is defined as the ratio of the number of males to the number of females in a population; a sex ratio greater than 1 implies that there are more men than women in a population (Table 1) [10, 11]. Since these demographic data were not available for Romania, a median of sex ratio (0.975) and age of women between 15 and 65 years (84%) were used. Several efforts were made to contact the industry in order to estimate the number of implants sold in each country, with special regards to brands and texture types. However, due to insufficient response rates, we decided that the report would not benefit these inconclusive data. Due to the lack of accurate sales data in each European country, an estimation of the prevalence proportion was calculated with the assumption that 3% of women older than 15 years are living with implants in line with Dutch data [12].

Morbidity refers herein to the number of persons with a confirmed histopathological BIA-ALCL diagnosis. Measures of morbidity frequently characterize the number of persons in a population who become ill (incidence) or are ill at a given time (prevalence). Incidence and prevalence were reported, but prevalence proportion requires sales rates of all manufacturers in the listed countries.

### Statistics

A one-way ANOVA was used to analyze four repeated measures of confirmed BIA-ALCL cases for each country. Results with a p-value below 0.05 were considered to be statistically significant.

## Results

Data from E(A)SAPS survey of 48 European countries are listed in Table 1. Of the 48 European countries, 33 returned the questionnaire twice annually. Countries in Table 1 showed the actual sex ratio for 2020 as ranging between 0.93 and 1.01.

**Fig. 1:**
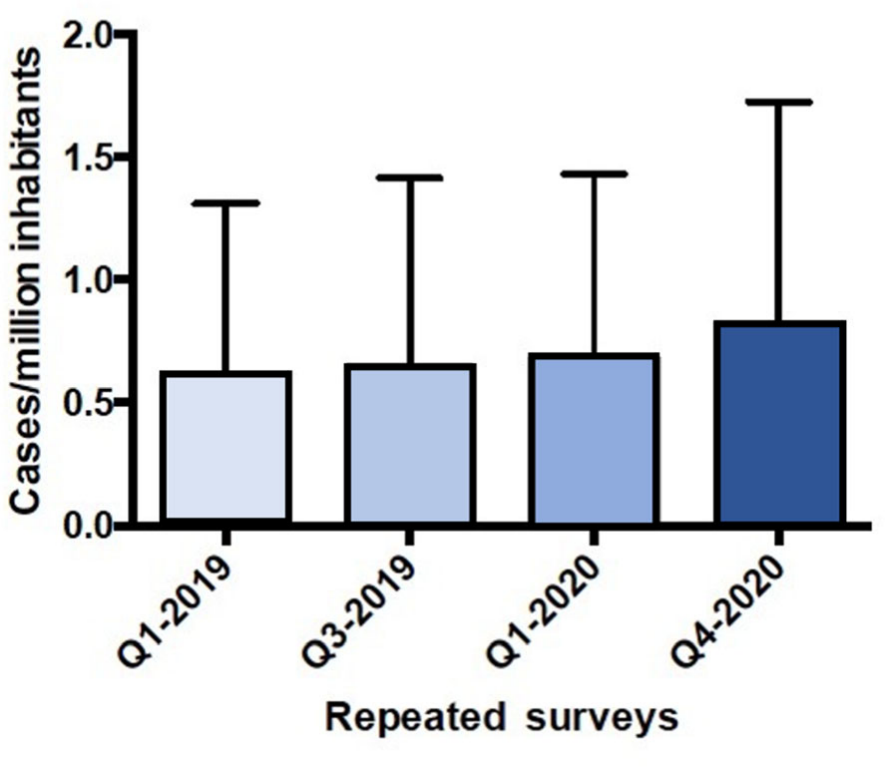
Reported number of BIA-ALCL cases per million inhabitants for the four timepoints of the conducted survey, presented as mean and standard deviation of all countries reporting cases

The number of reported BIA-ALCL cases significantly increased from a total of 305 in April 2019 to 434 in November 2020 (p<0.001) (Fig. 1). Fourteen out of the 434 patients had died of the disease at the latter time point. Ten countries reported no cases of BIA-ALCL. The total response rate was 69%, as 15 countries did not respond to the survey (Table 1).

**Fig. 2:**
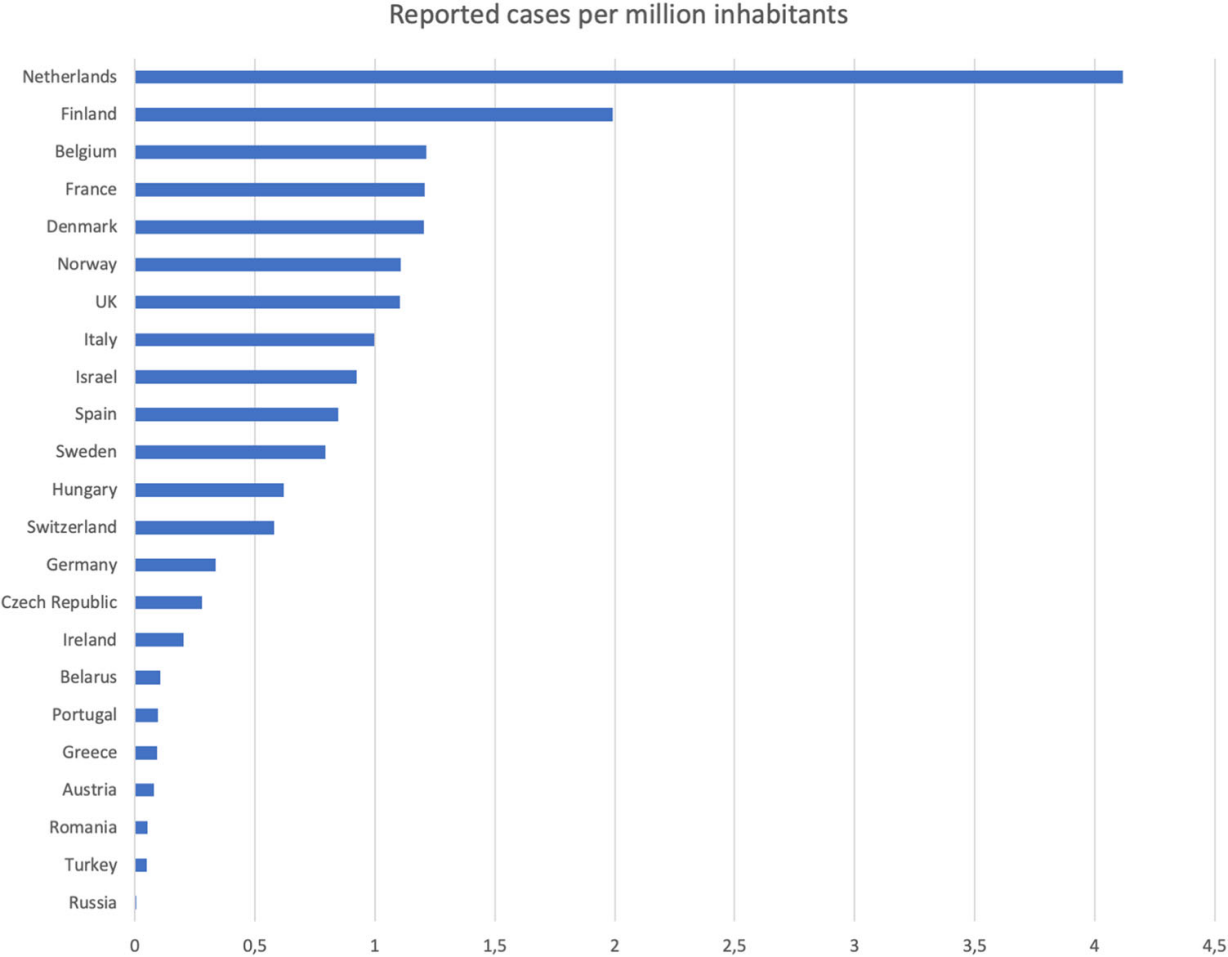
Reported BIA-ALCL cases per country and million inhabitants

Belgium, France, Germany, Italy, Netherlands, Spain and UK reported the highest total numbers (more than 10) of confirmed BIA-ALCL cases from 2019 to 2020 (Table 1). A large variation in the number of cases per million inhabitants was noted as demonstrated in the waterfall diagram in Fig. 2. The Netherlands had the highest rate (4.12) followed by Finland (1.99). Several countries had registered almost one case per million inhabitants, whereas the remaining countries reported significantly lower numbers. However, the reported rate for Russia was excluded from the above-mentioned rate since it was considered to be an outlier (0.0069) with a wide range from the following countries which had the lowest reported rates, i.e., Turkey (0.048), Romania (0.052) and Austria (0.078).

Based on current data in the literature, the proportion of women living with breast implants is reportedly 3 % in the Netherlands ^12^. Under this assumption, the prevalence was calculated for each country. The prevalence proportion, based on the assumption of 3%, displayed a range from 0.02 (Austria) to 0.33 (Netherlands) of BIA-ALCL cases per million women above the age of 15. Countries reporting existing registers are highlighted in first column (Table 1).

Five out of the 33 participating countries stated difficulties in accessing diagnostic facilities for analysis of CD 30 and ALK. Four out of these five countries had no or very low numbers of BIA-ALCL, apart from Spain which reported 40 BIA-ALCL cases, despite restricted accessibility to diagnostic investigations in some local areas.

In a majority of the countries, responding, the use of textured implants is still allowed. France is the only country with a ban on all textured implants. Bans of macrotextured implants have been implemented in eight countries according to the survey. In parallel, Allergan recalled Biocell^®^ implants 2019, which has practically prohibited the use in all European countries.

## Discussion

The current study identified 434 confirmed cases of BIA-ALCL and 14 deaths from BIA-ALCL in 33 participating European countries up to November 2020. Generated data were secured by four consecutive surveys conducted by The European Association of Societies of Aesthetic Plastic Surgery E(A)SAPS. A significant increase in reported cases was noted over the 19-month study period. Interestingly, the reported cases per million inhabitants displayed a nearly 100-fold variation from 0.048 (Turkey) to 4.1 (Netherlands) cases per million inhabitants. Whether this reflects the respective countries reporting systems, a variation in access to accurate diagnostic testing for BIA-ALCL, genetic variations or the prevalence of high-risk implants among women in certain countries can only be speculated. Most likely the reason for the reported wide range is multifactorial. The value of the current study is the identified wide range of cases per million inhabitants reported from 33 European countries. The range further reflects the difficulty for surgeons to provide accurate information for patients, based on a true estimated risk of BIA-ALCL in specific countries. Incomplete sales data, together with medical tourism, further preclude absolute risk assessment. The exact incidence of BIA-ALCL, based on the total number of women living with breast implants in each European country, is unknown, and the current E(A)SAPS survey could therefore not generate conclusive data regarding this point.

Of note, the estimation of prevalence proportion presented in the current study does not take implant brands into consideration. It is therefore important to interpret the numbers with caution, since BIA-ALCL has mainly been associated with macro-textured breast implants. However, a combination of textured surfaces, bacterial contamination and genetic predisposition all seems to contribute to the development of BIA-ALCL [1]. Although regional variations have been described before, surface texture seems to have a critical impact, with texture grades 3 and 4 seeming to pose a higher risk than grades 2 and 1. Brody et al analyzed the risk of developing BIA-ALCL in 173 cases and found a variation from 1:2832 to 1:86029, depending on surface type [13]. Cordeiro at al further found that ten women, 1/354, developed ALCL after a median exposure of 11.5 years (range, 7.4-15.8 years) in a cohort of 3546 women followed prospectively after reconstruction with textured implants [14].To the best of our knowledge, it has not been mandatory or regulated by law that European countries should collect national data on events related to breast implants in validated, industry-independent quality registers. The exceptions to this statement are the UK(mandatory within the National Health Services), the Netherlands and Australia where it is mandatory to register breast implants. Registers in other countries are depending on the goodwill of professionals, both in the field of aesthetic and reconstructive surgery. Even without legal regulations, it is highly desirable that European plastic surgeons introduce a database for breast implants in order to collect large data for robust statistical analysis and co-work with international initiatives such as ICOBRA [15]. With special regard to the large number of countries still using textured implants according to the survey, it is extremely important to collect aggregated long-term data in a European breast implant database. The total number of cases in the current study is in line with a recent study by Santanelli et al, where 420 cases of BIA-ALCL were estimated, according to data collected by the EURAPS-CDSD, following the WHO definition and the NCCN guidelines for diagnosis (PMID: 30715173) in the EU-28 (numerator) [16]. It is worth noting that this number was generated through an estimation, where only 61% of the EU-28 population countries were described to have implemented specific measures to tackle BIA-ALCL and actively reported 382 cases (PMID: 33022037). The current study therefore serves as an adjunct to the literature by reporting the highest number of confirmed cases of BIA-ALCL in European countries so far. Nevertheless, underreporting by several countries most likely explains the large variation in BIA-ALCL cases per inhabitant. Implementation of specific measures therefore needs to be adopted. The Netherlands, with the highest number of cases per inhabitant, may serve as a role model, where The Dutch Breast Implant Registry (DBIR) was compared to the registration of BIA-ALCL in the Dutch Nationwide Network and Registry of Histo- and Cytopathology as a proof of concept [7]. Variations in the diagnosis of ALCL could also explain the variation across countries. It is unclear what the awareness of pathologists for this rare disease is when presented with tissue after capsulectomy. Also is unknown is how often surgeons sent capsules and periprosthetic fluid for pathology when performing capsulectomies.

Lastly, in some countries there are institutes who provide second opinions when there is doubt about the ALCL diagnosis, but this is not available in all European countries. We believe that the current paper has shed light on the necessity of a task force that coordinates all efforts to set correct diagnosis and further controls all data from different countries.

Limitations of this actual study imply the fact that the incidence of BIA-ALCL across European countries could not been shown as the total number of women living with breast implants in each European country is uncertain. Even with estimated sales rates for each country, the influence of medical tourism would still be a potential confounder. The generated data from the current E(A)SAPS questionnaires could also not identify specific implants used as multiple surgeries had been performed in several patients. In the future, it would be of interest to distinguish BIA-ALCL cases with only one implant placement in their history from those with multiple implant placements, The low response rate (69%) is also a limitation, However, the non-responding countries have a generally low population; Albania, Andorra, Bulgaria, Croatia, Holy See (Vatican City), Iceland, Liechtenstein, Luxembourg, Malta, Monaco, Montenegro, Poland, San Marino, Slovakia and Slovenia.

In conclusion, the current study displays a notable variation in the number of confirmed BIA-ALCL cases. Variations in diagnosis and reporting systems may explain the differences, but the influence of genetic variations and the prevalence of high-risk implants cannot be excluded [17]. Incomplete sales data, together with medical tourism, preclude an absolute risk assessment. Measures need be taken to standardize the collection of data in the European countries so that a realistic assessment of the risk of BIA-ALCL can be given in the future

**Table 1:** Number of reported BIA-ALCL cases and deaths together with the estimated prevalence of ALCL for the assumption that 3% of that selected women population between 15 and 65 years has breast implants 12 is presented per country. Countries with existing breast registers at the time of the last survey are described in the last column

Country	BIA-ALCL	Deaths	Total population (M)	Wmn ratio	Wmn 15- <65y	Wmn population (M)	Estimated prevalence	Register
France	81	5	67.8	0.96	82	34.59	95.2	Yes
United Kingdom	75	3	65.7	0.99	82	33.02	92.3	Yes
Netherlands	70	2	17.2	0.98	84	8.69	319.8	Yes
Italy	60	1	62.4	0.93	87	32.33	71.1	Yes
Spain	40	1	50.0	0.98	85	25.26	62.1	Yes
Germany	28	0	80.1	0.96	87	40.87	26.3	Yes
Belgium	14	0	11.7	0.97	83	5.94	94.7	No
Finland	11	0	5.5	1.00	86	2.75	155.0	No
Israel	8	0	8.7	1.01	73	4.33	84.4	No
Sweden	8	2	10.2	1.00	82	5.10	63.8	Yes
Denmark	7	0	5.8	0.97	83	2.94	95.5	No
Hungary	6	0	9.7	1.06	85	4.71	50.0	Yes
Norway	6	0	5.4	1.00	82	2.70	90.3	No
Switzerland	5	0	8.4	0.97	85	4.26	46.0	Yes
Turkey	4	0	82.0	1.01	76	40.80	4.3	No
Czech Republic	3	0	10.7	0.97	84	5.43	21.9	No
Austria	2	0	8.8	0.96	85	4.49	17.5	Yes
Russia	1	0	141.7	0.86	77	76.18	0.6	No
Greece	1	0	10.6	0.95	86	5.44	7.1	No
Portugal	1	0	10.3	0.96	86	5.26	7.4	No
Belarus	1	0	9.4	1.06	85	4.56	8.6	No
Ireland	1	0	5.1	1.00	79	2.55	16.5	No
Romania	1	0	21.3	0.97	84	10.78	3.7	No

Reporting 0 cases: Bosnia/Herzegovina, Cyprus, Estonia, Georgia, Latvia, Lithuania, Macedonia, Moldavia, Serbia, Ukraine

Not participating: Albania, Andorra, Bulgaria, Croatia, Holy See (Vatican City), Iceland, Liechtenstein, Luxembourg, Malta, Monaco, Montenegro, Poland, San Marino, Slovakia, Slovenia

*M* million inhabitants, *Wmn ratio* female/male population, *estimated prevalence* (cases per million women at risk)
